# Dosimetric Evaluation of Intensity-Modulated Radiotherapy, Volumetric Modulated Arc Therapy, and Helical Tomotherapy for Hippocampal-Avoidance Whole Brain Radiotherapy

**DOI:** 10.1371/journal.pone.0126222

**Published:** 2015-04-20

**Authors:** Yi Rong, Josh Evans, Meng Xu-Welliver, Cadron Pickett, Guang Jia, Quan Chen, Li Zuo

**Affiliations:** 1 Department of Radiation Oncology, The Ohio State University James Cancer Hospital, The Ohio State University Wexner Medical Center, Columbus, Ohio, 43210, United States of America; 2 Radiologic Sciences and Respiratory Therapy Division, School of Health and Rehabilitation Sciences, Biophysics Graduate Program, The Ohio State University College of Medicine, The Ohio State University Wexner Medical Center, Columbus, Ohio, 43210, United States of America; 3 Department of Radiation Oncology, University of Virginia Health System, Charlottesville, Virginia, 22903, United States of America; 4 Department of Physics and Astronomy, Louisiana State University, Baton Rouge, Louisiana, 70803, United States of America; NIH, UNITED STATES

## Abstract

**Background:**

Whole brain radiotherapy (WBRT) is a vital tool in radiation oncology and beyond, but it can result in adverse health effects such as neurocognitive decline. Hippocampal Avoidance WBRT (HA-WBRT) is a strategy that aims to mitigate the neuro-cognitive side effects of whole brain radiotherapy treatment by sparing the hippocampi while delivering the prescribed dose to the rest of the brain. Several competing modalities capable of delivering HA-WBRT, include: Philips Pinnacle step-and-shoot intensity modulated radiotherapy (IMRT), Varian RapidArc volumetric modulated arc therapy (RapidArc), and helical TomoTherapy (TomoTherapy).

**Methods:**

In this study we compared these methods using 10 patient datasets. Anonymized planning CT (computerized tomography) scans and contour data based on fused MRI images were collected. Three independent planners generated treatment plans for the patients using three modalities, respectively. All treatment plans met the RTOG 0933 criteria for HA-WBRT treatment.

**Results:**

In dosimetric comparisons between the three modalities, TomoTherapy has a significantly superior homogeneity index of 0.15 ± 0.03 compared to the other two modalities (0.28 ± .04, *p <* .005 for IMRT and 0.22 ± 0.03, *p <* .005 for RapidArc). RapidArc has the fastest average delivery time of 2.5 min compared to the other modalities (15 min for IMRT and 18 min for TomoTherapy).

**Conclusion:**

TomoTherapy is considered to be the preferred modality for HA-WBRT due to its superior dose distribution. When TomoTherapy is not available or treatment time is a concern, RapidArc can provide sufficient dose distribution meeting RTOG criteria and efficient treatment delivery.

## Introduction

Brain metastases occur in 25–45% of cancer patients [1]. Whole brain radiotherapy (WBRT) is a radiation modality most commonly used for patients with numerous brain metastases caused by various malignancies. WBRT is also utilized in prophylactic cranial irradiation for patients with small cell lung carcinoma [2]. Unfortunately, WBRT is associated with many side effects including development of dementia [3], cerebellar dysfunction [4], and deficits in neurocognitive functions, such as short term memory (i.e. verbal recall ability) as well as the ability to concentrate and learn. Other neurocognitive functions such as fine motor control and executive functions do not show the same declines [2, 5–10].

Learning, memory, and spatial-processing impairments in those who have undergone WBRT treatment are believed to be caused by hippocampal injury [11]. Radiation induced hippocampal injury can be linked to damage to a small compartment of stem cells in the hippocampus which are particularly sensitive to the negative side effects of WBRT. These stem cells or “neural progenitor cells” are located within the dentate gyrus, a component of the larger hippocampus [12], which is part of the greater limbic circuit which includes the fimbriae, fornices, amygdala, and parahippocampal gyrus [13, 14]. In rodents, a small amount of radiation to this area has been shown to cause cell death and a decrease in neurogenesis in the subgranular zone of the dentate gyrus [15–20]. Moreover, the decline in neurogenesis is dose-dependent, with a 62% reduction of new neural stem cells and a 97% reduction of overall neurogenesis in the hippocampus after one fraction of 10 Gy [21, 22]. Lastly, irradiation of the precursor cells in rat models alters differentiation of the cells into primarily glial tissue as opposed to neuronal tissue [2, 20, 23]. Such alteration in differentiation may in turn affect hippocampal related cognitive functions. Hence, it has been proposed that the hippocampus be spared during WBRT in order to minimize the neurocognitive decline in patients.

Although avoiding the hippocampus is desirable, it is important that the rest of the brain receives an adequate and homogenous dose for reducing the probability of cancer growth. One foreseeable problem with sparing the hippocampus can occur for patients with tumors located in their hippocampi, thus making HA-WBRT an unsafe alternative to WBRT. Gondi and colleagues (2010) investigated this potential problem and found that out of 371 patients with 1,133 total metastases only 8.6% (11.5% being the upper limit on the 95% interval of confidence) of patients presented with a tumor inside the hippocampal avoidance region (the hippocampal avoidance region is defined as the hippocampi proper plus a 5mm expansion around the hippocampi) [24]. Therefore, with this estimation, HA-WBRT can be an effective treatment option for 91.4% of patients with brain metastasis (lower limit of 88.5% in the 95% confidence interval) [24]. To further reduce this likelihood, and to irradiate as much of the whole brain as possible, it has been suggested that the hippocampal avoidance region should be focused on the dentate gyrus and the cornus ammonus only (instead of the entire limbic circuit) [2, 17, 19].

### RTOG 0933

RTOG 0933 is a phase II trial studying the effectiveness and efficacy of hippocampal avoidance during WBRT for brain metastases. Stringent dose criteria in this protocol require a high level of dose modulation and precise delivery. Furthermore, the anatomical location of the hippocampal avoidance region in the brain makes optimization very difficult since the main organ-at-risk (OAR) is completely surrounded by the planning target volume (PTV). Helical TomoTherapy (TomoTherapy or “HT”) has been the first choice for the HA-WBRT treatment, due to its capability in achieving superior dose conformity and homogeneity [2]. More studies showed the feasibility of delivering an adequate dose using other more commonly available radiotherapy modalities, such as multi-beam intensity modulated radiotherapy (IMRT) and multi-arc volumetric modulated arc therapy (RapidArc) [2, 11]. Gondi and colleagues (2010) have compared HT and IMRT in terms of Hippocampal dose reduction, PTV coverage, and homogeneity [25]. We built on this work by expanding the number of patient datasets from 5 to 10, evaluating delivery efficiency, and also reviewing RapidArc as an additional treatment modality. This is the first investigation regarding effectiveness of these three modalities on the same patient data set. We aim to evaluate the dosimetric differences among these modalities in delivering HA-WBRT following the RTOG 0933 criteria.

## Methods

### Patient Population, CT (computerized tomography)/MRI (magnetic resonance imaging) Scans, Contouring and Planning Constraints

A total of ten patients were selected, anonymized and received HA-WBRT following the RTOG protocol 0933 requirements and IRB protocol (#2010C0078) at Ohio State University and the approved procedure (IRB-HSR #17875) at the University of Virginia Health System. This study was approved by the umbrella IRB-Ohio State University Cancer Institutional Review Board and the University of Virginia IRB for Health Science Research. Patient data were anonymized by the first and sixth authors. We used this umbrella IRB for all retrospective studies addressed in this paper. The IRB protocol number is 2010C0078. Since this was a retrospective study, all patient records were anonymized and de-identified prior to analysis. Therefore, patient consent was waived. The ethics committees/IRBs did approve the umbrella procedure. In addition, this research also involved previously collected data as part of the improvement project in which there was no intervention with a patient. All HIPAA identifiers were removed. Therefore, this study can be regarded as IRB exempt. Ten patients were selected for this comparison and as is shown later in the manuscript, this sample size is considered appropriate due to the minimal patient-to-patient anatomic and dosimetric variability. All patients had their CT simulation images fused with three-dimensional spoiled gradient (3D-SPGR) axial MRI scans (with 1.25 mm slice thickness). Hippocampus contours were drawn based on the 3D-SPGR MRI scans by the attending physicians strictly following the RTOG 0933 contouring guidelines. Contours for targets and other normal structures included the brain, brainstem, cord, eyes, lenses, chiasm, and optic nerves. The PTV consisted of the whole brain minus the hippocampi with a 5mm expansion around them. Following the RTOG guidelines, the treatment prescription is to deliver 30 Gy over the course of 10 fractions to the whole brain minus the hippocampi PRV. The RTOG 0933 acceptable compliance criteria for target and normal tissue planning doses are as follows:

1. At least 95% of brain volume receives 30 Gy (V_30Gy_ > 95% PTV)
2. 2% of the target volume receive 37.5 Gy or less (D_2%_ ≤ 37.5 Gy)
3. 98% of the target volume receive 25 Gy or more (D_98%_ PTV ≥ 25 Gy)
4. Minimum dose to the hippocampi (D_min_ = D_100%_) be ≤ 10 Gy
5. Maximum dose to the hippocampi be ≤ 17 Gy
6. Maximum dose to optic nerves & chiasm be ≤ 37.5 Gy

### Treatment Planning

Treatment plans were generated using 6 MV photons beams in three different planning and delivery modalities for comparison: step-and-shoot IMRT using the Pinnacle^3^ treatment planning system (Philips, Fitchburg, WI), TomoTherapy (Accuray Inc., Madison, WI), and RapidArc using the Eclipse planning system (Varian Medical Systems, Palo Alto, CA). While there are differences in the dose calculation algorithms for each of the three planning systems used in this study, such difference is minimal relative to the dosimetric differences from the delivery modalities investigated in this work.

Three planners generated treatment plans for all ten patients in accordance with the protocol compliance criteria as listed above. Each planner independently generated plans for only one modality based on their area of expertise. To help ensure a non-biased comparison for this study, each planner generated plans without prior knowledge of the other modalities’ plan quality. Below we include detailed planning guidance used for each modality in this study.

#### Linac Based IMRT Planning

Pinnacle^3^ version 9.0 m was used for LINAC-based step-and-shoot fixed field IMRT planning. RTOG protocol 0933 includes recommended planning approaches for linac-based IMRT planning (RTOG 0933 Appendix 8). Beam arrangement 1 was utilized which specifies nine non-coplanar beams with seven unique couch angles. On the Varian TrueBeam linear accelerator used for clinical HA-WBRT treatment delivery, the large size of the whole brain target necessitated beam splitting, which added to the overall treatment time. The beam model used for these plans has been fully commissioned and is in clinical use for HA-WBRT treatments at the University of Virginia. RTOG 0933 protocol recommended planning objectives (0933 Appendix 8) were used for inverse optimization using the direct machine parameter optimization (DMPO) algorithm.

#### TomoTherapy Planning

TomoTherapy plans were generated using a research version of the TomoTherapy planning station (NCCR release). This version utilizes a non-voxel based broad-beam optimization and dose calculation algorithm [26, 27] implemented on a graphics processing unit. The accuracy of the new algorithm has been well validated [28, 29]. To maximize sparing of the hippocampi while preserving target coverage, a 1.0 cm jaw width was employed. The pitch was selected as 0.215 and the modulation factor was set at 2.0. Following planning recommendations in RTOG 0933 appendix 8, complete blocks were used for lenses and directional blocks were used for eyes. Plans were prescribed such that 95% of the whole-brain PTV received the prescription dose of 30 Gy.

#### RapidArc Planning

The Varian RapidArc technique delivers single or multiple volumetric modulated arcs (VMAT) with varying gantry speed, dose rate, and MLC leaf travelling speed, to achieve optimal target coverage. For this study, two coplanar arcs (couch = 0°) at collimator 30° and 330° were used for each case. The Progressive Resolution Optimizer (version 10.0.28) was used for VMAT optimization.

### Dosimetric Plan Comparison

All 10 common patient CT datasets and structures and the 3 dose distributions for each patient were imported into Varian’s Eclipse treatment planning software for comparison of specific dosimetric metrics between the three modalities. Dose metrics according to the RTOG 0933 protocol compliance criteria were extracted. For the PTV, the volume receiving more than 30 Gy (V_30Gy_) and the minimum dose covering 98% of the volume are used to assess coverage. The dose delivered to the hottest 2% of the PTV (D_2%_) is used to determine the number of hotspots in the treatment plan. A homogeneity index (HI) was also calculated for each plan, where:

$$\begin{array}{l} \text{HI = }\frac{D_{2\%}-D_{98\%}}{D_{median}} \end{array}$$

HI quantifies dose homogeneity within the target volume. Minimizing HI is recommended by the International Commission on Radiation Units and Measurements [25]. D_median_ is defined as the median dose of the target volume. Smaller HI values closer to 0 indicate superior homogeneity, while larger values closer to 1 indicate inferior homogeneity [3]. Dose homogeneity is an important index in evaluating the plan quality, as largely heterogeneous dose distributions can result in detrimental effects on brain functions. The normal tissue doses extracted for comparison in this study include minimum and maximum doses to the hippocampus; maximum doses to the optic nerves, chiasm, and lenses, and the mean dose to the eyes.

### Delivery Time Comparison

The amount of time needed to deliver a single fraction of HA-WBRT was also recorded for all three modalities. In this work, the delivery time is defined as the time elapsed between the moments of the first beam-on to the end of the last beam-off and does not include pre-treatment patient setup and daily imaging procedures. Treatment delivery time was measured during “QA” delivery of the calculated plans.

### Statistical Analysis

Statistical comparisons between the three modalities’ treatment plans were performed using a one-way Analysis of Variance (ANOVA) and the Least Significant Distance (LSD) post-hoc tests using the SPSS Version 21 statistical software (IBM, USA). *p* values of < .05 were considered to be statistically significant.

## Results

Average dosimetric values for the 10 patient datasets (mean ± SD) are reported in Table 1. The table lists evaluated dosimetric metrics for the target and normal tissues (first and second column) with respect to each modality (first row). The second column includes RTOG 0933 dose compliance criteria, where available. The treatments for all patients were found to be in compliance with the RTOG 0933 protocol dosimetric criteria.

**Table 1:** Average dosimetric values across different brain structures under three types of treatments (Tomo, IMRT and RapidArc).

Structure	Dosimetry Metric (Protocol Criteria)	TomoTherapy	Step & Shoot IMRT	RapidArc
**PTV**	V_30Gy_(> 95%)	94.4% ± 0.6%	94.8% ± 0.3%	95.5% ± 0.8%
	D_2%_(≤ 37.5 Gy)	32.2 ± 0.6 Gy	36.3 ± 0.6 Gy	33.9 ± 0.4 Gy
	D_98%_≥(25 Gy)	27.5 ± 0.5 Gy	27.0 ± 0.9 Gy	26.8 ± 0.9 Gy
	HI	0.15 ± 0.03	0.28 ± 0.04	0.22 ± 0.03
**Hippocampus**	D_100%_(D_min_) ≤ 10 Gy	8.0 ± 0.3 Gy	8.7 ± 0.2 Gy	8.6 ± 0.3 Gy
	D_max_ ≤ 17 Gy	15.1 ± 0.8 Gy	14.9 ± 0.9 Gy	13.6 ± 1.3 Gy
**Optic Nerves & Chiasm**	D_max_ ≤ 37.5 Gy	33.9 ± 1.1 Gy	36.6 ± 0.5 Gy	34.4 ± 0.7 Gy
**Eyes**	D_max_	8.5 ± 0.9 Gy	8.8 ± 0.9 Gy	21.0 ± 3.3 Gy
	D_mean_	4.4 ± 0.4 Gy	5.6 ± 0.3 Gy	10.5 ± 2.1 Gy
**Lenses**	D_max_	3.3 ± 0.3 Gy	5.1 ± 0.3 Gy	8.1 ± 1.6 Gy
**Approximate Delivery Time**		18.0 min	15.0 min	2.5 min

Each value was calculated based on the data from 10 patients. Values are expressed as mean ± SD.

### PTV, Hotspots, Minimum Target Dose and Homogeneity Index

The statistical analysis of pairwise comparisons among IMRT, RapidArc and TomoTherapy are shown in Table 2. Comparing the PTV coverage across modalities, IMRT provided an average of 94.8% for V_30Gy_, which was comparable to TomoTherapy (94.4%, *p* = .17). The RapidArc plans, on the other hand, provided an average of 95.5% for V_30Gy_, which was significantly higher than the TomoTherapy (*p* < .001) and IMRT result (*p* < .05). In the evaluation of hotspots, the average D_2%_ for IMRT (36.3 Gy) was inferior to RapidArc (33.9 Gy) (*p* < .005) and they both showed a significantly higher D_2%_ compared to TomoTherapy (32.2 Gy, *p* < .005). In terms of the minimum target dose criteria, D_98%_, both TomoTherapy (27.5 Gy) and RapidArc (26.8 Gy) were comparable to IMRT (27.0 Gy). The only statistically significant difference was found between TomoTherapy and RapidArc (*p* < .05). Helical TomoTherapy had a mean homogeneity index (HI) of 0.15, compared to 0.22 for RapidArc and 0.28 for IMRT. These findings indicate that the most effective target dose homogeneity was achieved by TomoTherapy, followed by RapidArc, and IMRT as the least effective in achieving dose homogeneity.

**Table 2:** Comparison of the three treatments (Tomo, IMRT and RapidArc) in terms of dosimetry metric and related *p* values.

Structure	Dosimetry Metric	*p* value		
		Tomo vs. IMRT	Tomo vs. RapidArc	IMRT vs. RapidArc
**PTV**	V_30Gy_	0.167	0.000**	0.015*
	D_2%_	0.000**	0.000**	0.000**
	D_98%_	0.130	0.036*	0.576
	HI	0.000**	0.000**	0.001**
**Hippocampus**	D_100%_ (D_min_)	0.000**	0.000**	0.596
	D_max_	0.726	0.004**	0.023*
**Optic Nerves & Chiasm**	D_max_	0.000**	0.218	0.000**
**Eyes**	D_max_	0.734	0.000**	0.000**
	D_mean_	0.032*	0.000**	0.000**
**Lenses**	D_max_	0.000**	0.000**	0.000**

Each *p* value was calculated based on the data from 10 patients;

* *p* < 0.05;

** *p* < 0.005 (*one-way ANOVA*, *LSD post-hoc test*).

### Hippocampal Avoidance, Optice Chiasm and Nerves

In terms of Hippocampal avoidance, both IMRT (8.7 Gy, *p* < .001) and RapidArc (8.6 Gy, *p* < .001) had a higher mean D_100%_ compared to TomoTherapy (8.0 Gy). However, these doses are still within the protocol’s acceptable criteria of 10 Gy. There was no significant difference between IMRT and RapidArc in D_100%_. Although the average hippocampal D_max_ doses of IMRT (14.9 Gy) was not significantly different from TomoTherapy (15.1 Gy), RapidArc had a significantly lower D_max_ (13.6 Gy) than both Tomotherapy (*p* < .001) and IMRT (*p* < .05). In comparing the maximum doses for optic nerves and chiasm, the average D_max_ of the IMRT (36.6 Gy) was significantly higher than both TomoTherap (33.9 Gy, *p* < .005) and RapidArc (34.4 Gy, *p* < .005), while RapidArc provided a comparable D_max_ (34.4 Gy, *p* = .22) to TomoTherapy.

### Eyes and Lenses

For eyes and lenses, structures not specifically mentioned in the RTOG 0933 protocol dosimetric compliance criteria, the IMRT plans seemed to provide comparable average D_max_ of 8.8 Gy (*p* > .05) but a higher D_mean_ of 5.6 Gy (*p* < .05) for the globes as compared to TomoTherapy (D_max_ = 8.5 Gy; D_mean_ = 4.4 Gy). Furthermore, RapidArc (D_max_ = 21.0 Gy; D_mean_ = 10.5 Gy) showed a much higher D_max_ and D_mean_ globe values as compared to TomoTherapy (*p* < .005) and IMRT plan (*p* < .005). For lenses, RapidArc (D_max_ = 8.1 Gy) has significantly larger D_max_ than IMRT (D_max_ = 5.1 Gy, *p* < .005), and they both display higher values compared to TomoTherapy (D_max_ = 3.3 Gy, *p* < .05). These differences in globe and lens doses are likely attributable to differences in the relative importance of these non-protocol specified structures in the optimization by the individual planners for each modality. The 0933 protocol does not set any dose limits for these structures, and while appendix 8 in the protocol gives recommended planning constraints for static-field IMRT and TomoTherapy, there are no recommendations for RapidArc planning. In addition, TomoTherapy plan optimization allows for full or directional blocks to be employed to reduce the dose to these structures.

### Delivery Time

Regarding treatment delivery time, the HT technique took the longest time in treatment delivery (18 minutes) averaging over 10 patients, comparing to 2.5 minutes for RapidArc and 15 minutes for IMRT. It is noted that the delivery time was recorded from the moment of first beam-on to the end of the last beam-off and does not include any differences in pre-treatment patient setup and daily imaging procedures. The high TomoTherapy delivery time is attributable to the narrow collimation and low pitch while the high IMRT treatment time is attributable to the 7 unique couch angles requiring a therapist to enter the room multiple times.

### Dose Distribution

**Fig 1:**
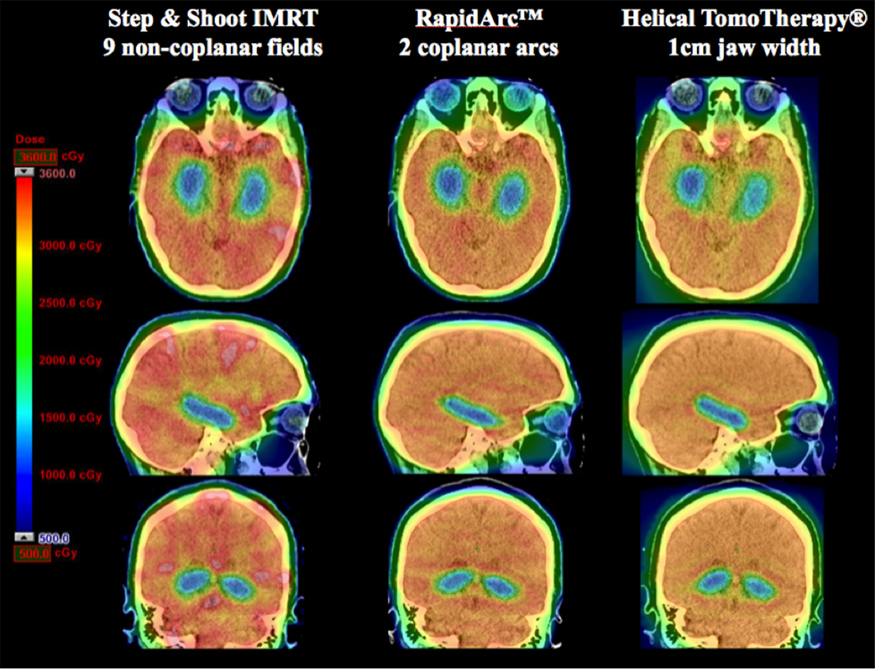
Dose-color-wash comparison for IMRT, RapidArc and Tomotherapy modalities. This figure depicts a representative patient’s dose-color-wash comparison for each of the treatment modalities. This patient was chosen because their dosimetric parameters most closely matched the mean value of the 10 patient data sets.

Fig 1 depicts dose distribution color washes from the three treatment modalities for one representative patient case in our study. The prescription dose of 30 Gy for the PTV is presented in the chart by a yellow hue. The IMRT dose distribution depicts several large, red spots that indicate hotspot regions in excess of 36 Gy (120% of the prescription dose). A total of 52 cc’s of this patient’s brain received a dose greater than 36 Gy for the IMRT plan, while the RapidArc and TomoTherapy treatment modalities had no portions of the brain receiving more than 36 Gy. Fig 1 also illustrates the superior HI of Tomotherapy and RapidArc plans as can be seen by their homogenous yellow hue and lack of bright red spots. The hippocampal avoidance region is adequately spared by all modalities. This can be seen by the homogenous, dark-blue hue in all of the hippocampi as shown in Fig 1.

### Dose Volume Histograms

**Fig 2:**
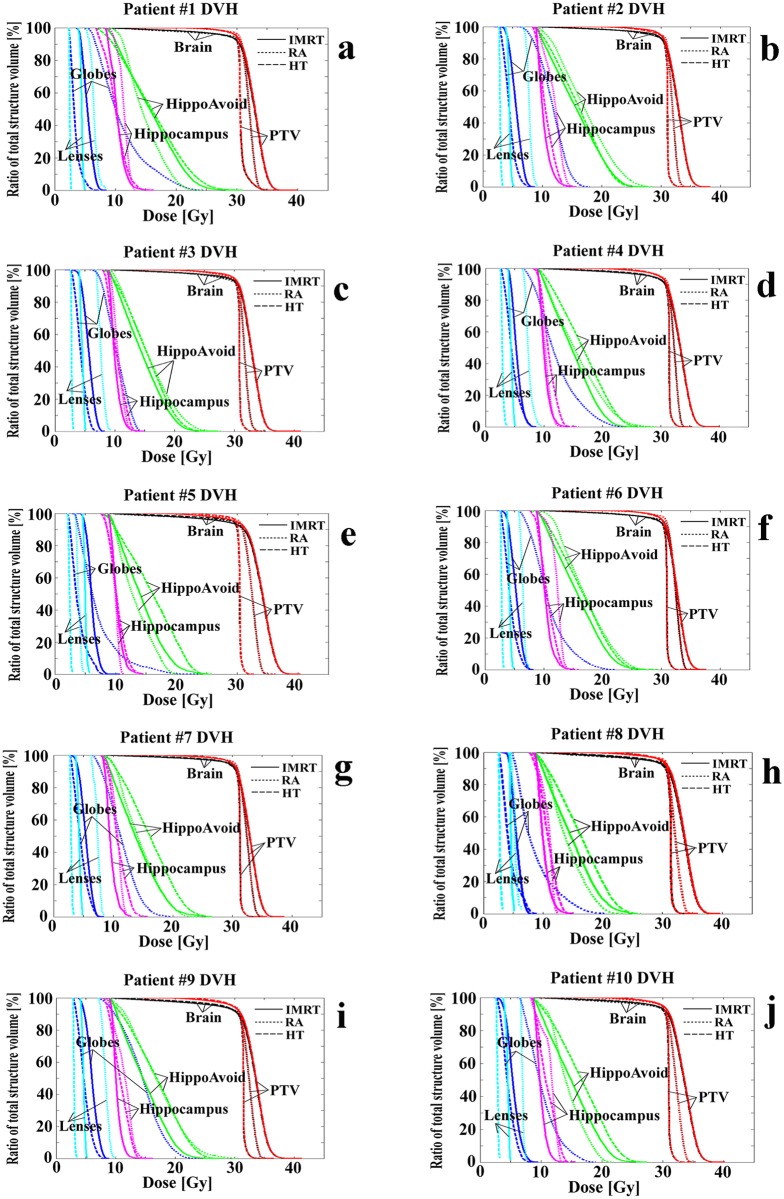
Dose-volume-histograms for ten patient data set. Dose volume histograms (DVH) for all 10 patients in our study with a planning evaluation of step-and-shoot IMRT, RapidArc, and TomoTherapy for the brain.

Fig 2 displays dose volume histograms (DVHs) for PTV, brain, lenses, globes, hippocampus, and hippocampi avoidance structures for all 10 patient data sets. The horizontal axis represents the dose (Gy) received, while the vertical axis lists the percentage of the total structure volume that receives the specified dose. The various modalities are represented by different line styles, i.e. solid lines for IMRT, dotted lines for RapidArc (VMAT), and dashed lines for TomoTherapy. Viewing the DVHs for all ten individual patient cases visually supports the conclusions of the dosimetric summary data presented in Table 1. TomoTherapy exhibits the steepest PTV DVH slope for all ten cases, followed by RapidArc and IMRT, which is consistent with the HI comparison.

## Discussion

To summarize the results, while all three modalities met RTOG 0933’s basic dosimetric compliance criteria, we found that TomoTherapy provided the most homogeneous target dose, (mean HI = 0.15) and RapidArc was the fastest method at 2.5 minutes on average for delivery time. In terms of HI and delivery time, we found step-and-shoot IMRT to be inferior on both accounts compared to the other two methods. We found that the patient-to-patient dosimetric variability is minimal, as shown by the small standard deviations in Table 1. Consequently, the dosimetric differences amongst treatment modalities were found to be statistically significant, which suggests our cohort of 10 patients was sufficient for this comparison.

Major differences in the radiation administration in each of the treatment plans led to significantly different delivery times. The relatively slow delivery time in TomoTherapy (18 minutes on average) can be attributed to the smaller 1 cm jaw width of the TomoTherapy treatment modality. The high delivery time in the IMRT plan (15 minutes on average) can be linked to the 7 couch kicks recommended by the RTOG 0933 protocol, while each kick required an experienced therapist to enter the room to make the couch adjustment. The coplanar double arc technique in RapidArc was shown to be by far the most time efficient (2.5 minutes on average) since the treatment was administered at a single couch angle, requiring only one patient setup by the therapists. This protocol reduced the delivery time in RapidArc to only 11–15% of the other two treatment modalities. The fast treatment not only increases the throughput, but also reduces patient discomfort and the possibility of patient motion.

There have been efforts in increasing the quality of TomoTherapy modality while lowering its delivery time. The high dosimetric efficacy of TomoTherapy can be attributed to the use of 1.0 cm jaw width as well as more degrees of freedom that Accuracy Helical TomoTherapy can provide for intensity modulation. On the other hand, these factors greatly reduce the utilization of the radiation produced. As a result, TomoTherapy has the longest treatment time among the three modalities studied. Note that even though Accuracy TomoTherapy has recently launched a new feature called “TomoEDGE” that can reduce treatment time by half for some cancer sites [30], this feature does not benefit the WBRT case presented here. The TomoEDGE reduces treatment duration by choosing a larger jaw width. However, the larger jaw width would produce wider dose penumbra which would jeopardize hippocampus sparing. It has been illustrated [30] that, since the hippocampus is centrally located in the PTV, the TomoEDGE’s running-start-stop delivery cannot help with the dose penumbra in this complex geometry.

In terms of the RTOG0933 ROI guidelines specifically, TomoTherapy outperforms RapidArc and IMRT in terms of 1) achieving a superior homogeneity index; 2) achieving a lower minimum dose (D_min_) in hippocampus region; 3) minimizing dose to the optic nerves and chiasm; 4) reducing average dose imparted to the eyes; 5) and reducing the maximum dose to the lenses. Moreover, TomoTherapy is as effective as IMRT in its ability to reduce maximum dose to the eyes. On the other hand, RapidArc outperforms the other modalities in minimizing the maximum dose to the hippocampus, and reducing overall delivery time. Step and Shoot IMRT was not found to be superior to the other modalities in any of the dosimetric parameters evaluated in the present study.

It is important to note that while our results show statistically significant differences in dose amongst treatment modalities, the absolute difference in dose may not be clinically significant. For example, It remains unknown that if it is preferable to have the lowest minimum dose, mean dose, or maximum dose to the hippocampus. In addition, it is still unknown that if the technically achievable doses to the hippocampus ranging from 8 Gy to 17 Gy are sufficient to spare enough stem cells to reduce neurocognitive toxicities from whole brain RT. These are questions that RTOG 0933 results and correlated analysis might be able to answer. Although our knowledge of the dose-toxicity relationship for individual organs is still evolving, higher radiation doses will always lead to higher cell kill (excluding the radiation hormesis hypothesis for very low doses). Ultimately, it is up to the physician to determine the acceptable dose limits for a given treatment; this paper highlights that the choice of treatment modality is a relevant point of discussion for HA-WBRT.

The findings in this investigation differ from studies by Gondi and colleagues [25] in several ways. Gondi and colleagues investigated the efficacy of only the TomoTherapy and the IMRT methods and found both treatment modalities to be equally effective for WBRT. However in this study, TomoTherapy was significantly more effective in reducing the minimum hippocampal dose than static field IMRT. Our findings are consistent with previous studies, which showed Helical TomoTherapy demonstrates a better capacity for selective sparing of tissue compared to IMRT [31]. These results will allow us to better discern the effectiveness of each modality in terms of WBRT side- effect mitigation. Additionally, our future work will include investigation of other techniques such as head tilting for HA-WBRT setup. Other reports in the literature show the tilted head geometry may reduce treatment time for the static-field IMRT modality [32], though it is unknown if it would also improve the dosimetry compared to the current data.

## Conclusion

For HA-WBRT, TomoTherapy offers superior dosimetric advantages over its competitors, especially in target dose homogeneity, minimum hippocampus dose and dose sparing for lenses and eyes. In this study, RapidArc plans provide the lowest maximum dose to hippocampus and the least amount of time needed for administration of the treatment.
